# Supplementary figures and images for: Influence of learning strategy on response time during complex value-based learning and choice
Source: PLoS One. 2018 May 22;13(5):e0197263. doi: 10.1371/journal.pone.0197263 (PMC5963802; doi:10.1371/journal.pone.0197263)

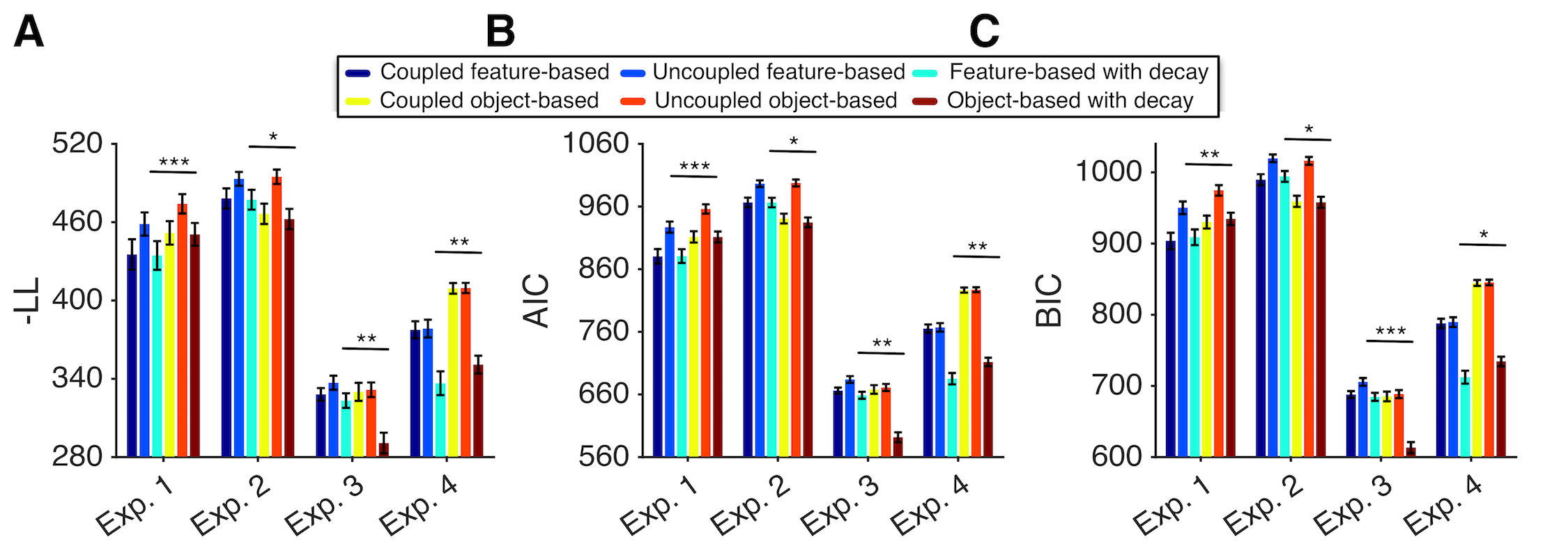

Supplement: S1 Fig — Panels (A-C) plot the goodness-of-fit measures in terms of the, negative log likelihood (-LL), Akaike information criterion (AIC), and Bayesian information criterion (BIC), respectively. The goodness-of-fit values are computed by averaging over all subjects (mean ± s.e.m.) separately for three feature-based RLs and their object-based counterparts and for Experiments 1 to 4. The significance level of the comparison between each model that provides the best fit in a given experiment and its object-based or feature-based counterpart is coded as: 0.01 < P < 0.05 (*), 0.001 < P < 0.01 (**), and P < 0.001 (***). (TIFF) [file pone.0197263.s001.tiff]
