# Supplementary material for: Influence of learning strategy on response time during complex value-based learning and choice
Source: PLoS One. 2018 May 22;13(5):e0197263. doi: 10.1371/journal.pone.0197263 (PMC5963802; doi:10.1371/journal.pone.0197263)
Supplement: S1 Table — Reported values are mean±std for each experiment. During Experiments 1 and 4, more subjects adopted feature-based learning whereas during Experiments 2 and 3 more subjects adopted object-based learning. The differences between the percentages of trials identified as feature-based and object-based were compatible with the overall adopted learning strategy in each experiment. (DOCX) [file pone.0197263.s002.docx]

| Experiment  (no. subjects) | % Feature-based subjects (# subjects) | % Feature-based  trial | % Equivocal trials | % Object-based trials |
| --- | --- | --- | --- | --- |
| Exp. 1 (n = 43) | 88 (n = 38) | 40.2±9.2 | 27.9±12.3 | 31.9±7.2 |
| Exp. 2 (n = 21) | 24 (n = 5) | 38.3±8.7 | 18.0±7.3 | 43.7±10.4 |
| Exp. 3 (n = 27) | 30 (n = 8) | 32.7±7.5 | 17.1±5.8 | 50.1±11.9 |
| Exp. 4 (n = 25) | 76 (n = 19) | 43.1±6.9 | 23.3±3.3 | 33.1±7.4 |

**S1 Table**
