# Supplementary material for: Influence of learning strategy on response time during complex value-based learning and choice
Source: PLoS One. 2018 May 22;13(5):e0197263. doi: 10.1371/journal.pone.0197263 (PMC5963802; doi:10.1371/journal.pone.0197263)
Supplement: S2 Table — Reported are the p-values (two-sided signed-rank test) for comparisons of the average RT between a given pair of trials (across subjects) depicted in Fig 6. (DOCX) [file pone.0197263.s003.docx]

|  | abs&rew vs.  abs&unrew | pres&rew vs.  pres&unrew | pres&rew vs.  abs&rew | abs&unrew vs.  pres&unrew | pres&rew vs.  abs&unrew | abs&rew vs.  pres&unrew |
| --- | --- | --- | --- | --- | --- | --- |
| Exp. 1 | 5.8*10^-7^ | 0.0425 | 0.4688 | 3.4*10^-8^ | 1.8*10^-5^ | 0.0490 |
| Exp. 2 | 0.0078 | 0.0355 | 0.9861 | 6.9*10^-5^ | 0.0355 | 0.0046 |
| Exp. 3 | 2.6*10^-5^ | 0.0088 | 0.3488 | 3.5*10^-5^ | 1.0*10^-3^ | 0.0023 |
| Exp. 4 | 0.0049 | 0.0087 | 0.6377 | 3.0*10^-4^ | 0.1155 | 0.0074 |

**S2 Table**
