# Supplementary material for: Influence of learning strategy on response time during complex value-based learning and choice
Source: PLoS One. 2018 May 22;13(5):e0197263. doi: 10.1371/journal.pone.0197263 (PMC5963802; doi:10.1371/journal.pone.0197263)
Supplement: S3 Table — Reported are the average values of BIC over all subjects (mean±s.e.m.) and p-values for comparisons of BIC values between each model and its object-based or feature-based counterparts (two-sided Wilcoxon signed-rank test). The overall best model (feature-based or object-based with decay) and its object-based or feature-based counterpart are highlighted in cyan and brown, respectively. (DOCX) [file pone.0197263.s004.docx]

| Model | Coupled feature-based | Uncoupled feature-based | Feature-based with decay | Coupled object-based | Uncoupled object-based | Object-based with decay |
| --- | --- | --- | --- | --- | --- | --- |
| # pars. | 5 | 5 | 6 | 4 | 4 | 5 |
| Exp. 1 | 1008.4±22.4  (*p* = 0.43) | 1016.9±22.9  (*p* = 0.82) | 1014.6±23.0  (*p* = 0.49) | 1017.1±20.2 | 1036.1±21.0 | 1025.4±20.3 |
| Exp. 2 | 994.9±27.4 | 1026.5±25.3 | 993.8±28.1 | 989.2±25.3  (*p* = 0.62) | 1015.7±24.6  (*p* = 0.03) | 987.4±24.8  (*p* = 0.40) |
| Exp. 3 | 746.5±10.2 | 734.8±12.0 | 698.2±11.8 | 777.2±5.9  (*p* = 0.03) | 750.1±11.6  (*p* = 0.20) | 698.3±13.0  (*p* = 0.57) |
| Exp. 4 | 901.6±11.5  (*p* = 0.003) | 870.7±24.8  (*p* = 0.042) | 840.1±31.4  (*p* = 0.68) | 924.9±5.8 | 905.2±11.3 | 871.3±25.2 |
