# Supplementary material for: Influence of learning strategy on response time during complex value-based learning and choice
Source: PLoS One. 2018 May 22;13(5):e0197263. doi: 10.1371/journal.pone.0197263 (PMC5963802; doi:10.1371/journal.pone.0197263)
Supplement: S4 Table — We used a GLM to predict the normalized RT as a function of the estimated probability of reward of the two objects presented on a given trial (absolute difference in subjective reward probability), the trial number within a block of the experiment, the difference between BIC per trial (BICp) based on the best feature-based and object-based models (i.e., model-adoption index) for a given subject, and the reward outcome on the preceding trial. Reported values are the normalized regression coefficients (±s.e.m.), p-values for each coefficient (two-sided t-test), and adjusted R-squared for each experiment. No interaction term was statistically significant and thus, interactions terms are not reported here. (DOCX) [file pone.0197263.s005.docx]

| Regressor | Abs. difference in subjective reward prob. | Trial number | BIC_p_ (Ft) – BIC_p_ (Obj) | Reward outcome on prev. trial | R^2^ |
| --- | --- | --- | --- | --- | --- |
| Exp. 1 | -0.06±0.010  (*p* = 1.4*10^-9^) | -0.16±0.010  (*p* = 10^-16^) | 0.01±0.009  (*p* = 0.59) | -0.04±0.010  (*p* =1.7*10^-6^) | 0.030 |
| Exp. 2 | -0.02±0.008  (*p* = 3.5*10^-11^) | -0.10 ±0.008  (*p* = 10^-16^) | -0.02±0.008  (*p* = 0.004) | -0.04±0.008  (*p* = 4.7*10^-7^) | 0.012 |
| Exp. 3 | -0.12±0.014  (*p* = 10^-16^) | -0.10±0.014  (*p* = 2.7*10^-12^) | 0.01±0.014  (*p* = 10^-16^) | -0.06±0.014  (*p* = 1.8*10^-6^) | 0.028 |
| Exp. 4 | -0.04±0.007  (*p* = 10^-5^) | -0.24±0.007  (*p* = 10^-16^) | -0.01±0.007  (*p* = 0.97) | -0.05±0.007  (*p* = 3.5*10^-6^) | 0.062 |

**S4 Table**
